# Advances in non-apoptotic regulated cell death: implications for malignant tumor treatment

**DOI:** 10.3389/fonc.2025.1519119

**Published:** 2025-01-30

**Authors:** Yizheng Zhang, Shiqi Yi, Mingyuan Luan

**Affiliations:** ^1^ Department of Pathology and Neuropathology, University Hospital and Comprehensive Cancer Center Tübingen, Tübingen, Germany; ^2^ Department of Obstetrics and Gynecology, West China Second Hospital, Sichuan University, Chengdu, China

**Keywords:** regulated cell death, tumorigenesis, cancer treatment, pyroptosis, necroptosis, ferroptosis, parthanatos, cuproptosis

## Abstract

Cell death mechanisms are broadly classified into accidental cell death (ACD) and regulated cell death (RCD). ACD such as necrosis, is an uncontrolled, accidental process, while RCD is tightly regulated by specific signaling pathways and molecular mechanisms. Tumor cells are characterized by their ability to evade cell death and sustain uncontrolled proliferation. The failure of programmed cell death is a key contributor to tumor initiation, progression, and resistance to cancer therapies. Traditionally, research has focused primarily on apoptosis as the dominant form of RCD in cancer. However, emerging evidence highlights the importance of other non-apoptotic forms of RCD, such as pyroptosis, ferroptosis, necroptosis, and parthanatos, in tumorigenesis and treatment response. These pathways are gaining attention for their potential roles in overcoming therapy resistance. In this review, we will discuss the recent advances in the study of non-apoptotic cell death pathways in malignant tumors and explore their therapeutic implications, offering insights into new targets for cancer treatment strategies.

## Introduction

In 1972, John Kerr and colleagues coined the term apoptosis to describe a form of programmed cell death (PCD) in response to intrinsic pathological signals. PCD later evolved into the concept of RCD, which includes both pathologically induced and pharmacologically modulated cell death (1). RCD exhibits distinct morphological features, differentiating it from accidental cell death, such as necrosis. Since then, research on regulated cell death has grown exponentially (2). Over the past three decades, apoptosis has garnered significant attention from the scientific community, with its molecular mechanisms being relatively well elucidated. Apoptosis primarily occurs through two distinct pathways: the extrinsic and intrinsic mitochondrial pathways. The extrinsic pathway is typically regulated by death-related membrane receptors, such as FAS and TNFR, and is driven by initiator caspases, including CASP8 and CASP10 (3). In contrast, the intrinsic pathway is triggered by mitochondrial outer membrane permeabilization (MOMP), leading to the release of mitochondrial proteins that activate the initiator caspase CASP9 and downstream effector CASP3, a process tightly regulated by the BCL2 family of proteins (4, 5).

Cell death is a crucial biological process that regulates organismal development and maintains homeostasis (6). Defects and dysregulation of normal cell death signals promote tumor initiation and progression, which is a hallmark of malignant tumors (7). The role of apoptosis in tumor cell survival and how to target and induce apoptosis has been a major focus of antitumor drug development in recent decades (8, 9). Clinically, apoptosis-inducing drugs, including cytotoxic chemotherapies and targeted therapies, are widely used in the treatment of malignant tumors (10). However, due to the significant heterogeneity of tumors, some patients gradually develop reduced sensitivity or even primary resistance to anti-tumor treatments, severely affecting therapeutic efficacy (11, 12). Tumor cell resistance to apoptosis has been identified as a key mechanism behind drug resistance (13). Therefore, finding ways to effectively activate cell death pathways when apoptosis is inhibited represents a potential strategy for overcoming tumor resistance, though it remains a significant challenge.

In addition to apoptosis, various other forms of RCDs have been identified and extensively studied, including pyroptosis, necroptosis, ferroptosis, parthanatos, anoikis, autophagy-dependent cell death, entosis, mitotic catastrophe, lysosome-dependent cell death, disulfidptosis, cuproptosis and alkaliptosis (14, 15). RCD occurs in both physiological and pathological contexts, playing a critical role in maintaining cellular homeostasis. Dysregulation of these processes is frequently implicated in the development of various diseases, particularly cancer (16). Importantly, targeting RCD-associated proteins and pathways might offer a promising therapeutic approach for overcoming resistance to conventional treatments, providing new hope for patients who have developed resistance to standard therapeutic agents.

In this review, we will explore the diverse pathways of RCD, emphasizing their key features, mechanistic details, and significance in cancer treatment, particularly in relation to cancer progression and drug resistance. Additionally, we will analyze the intricate cross-talk between various RCD signaling pathways, highlighting their complex interactions in the cancer treatments. Furthermore, we will assess the therapeutic potential of targeting different forms of RCD as innovative strategies for overcoming drug resistance and enhancing treatment efficacy in cancer patients. These emerging approaches offer new insights and hope for improving clinical outcomes in cancer therapy.

## Pyroptosis

Pyroptosis, also known as inflammatory cell death, is a form of RCD driven by inflammasomes, and it exhibits distinct morphological features compared to apoptosis. Unlike apoptosis, pyroptosis does not involve significant DNA fragmentation but is characterized by notable nuclear condensation, pore formation in the plasma membrane, and cell swelling (17). Inflammasomes are cytoplasmic multiprotein complexes that are typically activated by external stimuli, such as lipopolysaccharides (LPS), and they play a crucial role in the release of interleukin family members (e.g., IL-1β, IL-18), formation of the adaptor protein ASC, and activation of pro-inflammatory caspases, which ultimately induce pyroptosis (18).

In the classical pathway, pyroptosis is mediated by caspase-1, whereas in the non-classical pathway, it is mediated by caspase-4, caspase-5, and caspase-11. Caspase-4 and caspase-5 mediate pyroptosis in human cells, while caspase-11 functions in murine cells. Activated caspase-1 cleaves pro-IL-1β and pro-IL-18 into their mature forms, which are then released extracellularly, triggering an inflammatory response (19). Concurrently, activated caspase-1 cleaves gasdermin D (GSDMD) into a 22 kDa C-terminal fragment (GSDMD-C) and a 31 kDa N-terminal fragment (GSDMD-N) (20, 21). The GSDMD-N fragment translocates to the plasma membrane, binds to the phospholipid bilayer, and forms transmembrane pores, leading to membrane rupture and cell lysis (22). Additionally, research has shown that pyroptosis can also be mediated by the caspase-8-GSDMD and caspase-3-GSDME pathways, indicating that in certain contexts, pyroptosis and apoptosis may occur simultaneously (23, 24).

**Figure 1:**
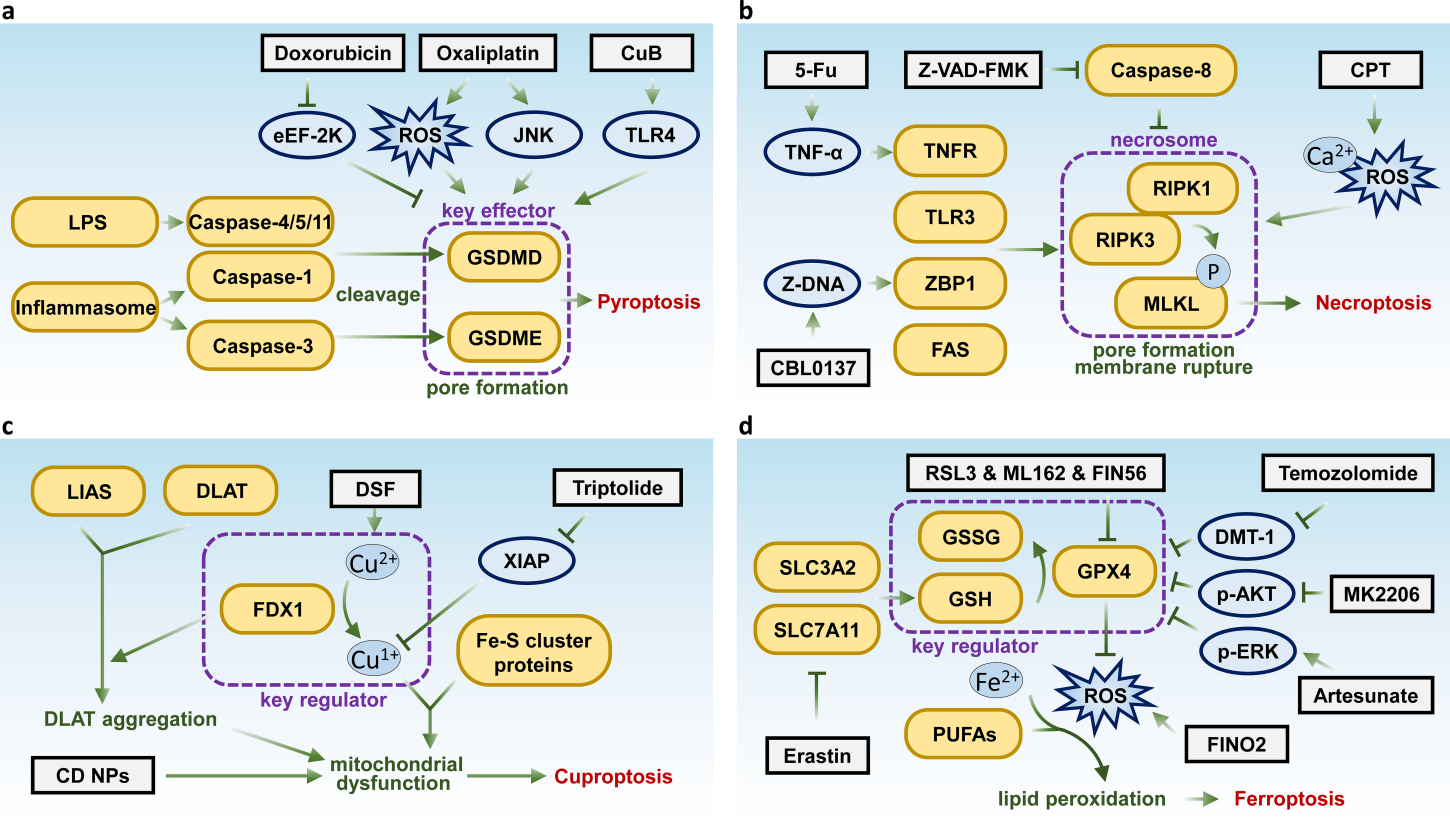
Key molecules and therapeutic targets in pyroptosis, necroptosis, cuproptosis, and ferroptosis. Schematic summarizing the key molecular pathways and therapeutic targets of **(A)** pyroptosis, **(B)** necroptosis, **(C)** cuproptosis, and **(D)** ferroptosis.

As a form of cell death, pyroptosis has the potential to inhibit tumor initiation and progression. Studies have shown that various chemotherapeutic agents, targeted therapies, and natural compounds can induce pyroptosis in a range of different tumors (25, 26). For instance, chemotherapeutic drugs such as doxorubicin, actinomycin D, bleomycin, paclitaxel, and cisplatin have been found to induce pyroptosis in lung cancer cells through the caspase-3-GSDME pathway (24, 27). Additionally, doxorubicin has been shown to induce pyroptosis in melanoma by inhibiting eukaryotic elongation factor-2 kinase (eEF-2K), which not only enhances the anti-tumor effects but also suppresses autophagy (28). Moreover, the third-generation platinum-based anti-cancer drug, oxaliplatin, has been reported to induce pyroptosis in colon cancer cells through elevated levels of reactive oxygen species (ROS) and activation of the JNK kinase, also via the caspase-3-GSDME pathway (29). Additionally, the small molecular compound cucurbitacin B (CuB) has been shown to inhibit non-small cell lung cancer both *in vitro* and *in vivo* by triggering pyroptosis through the TLR4/NLRP3/GSDMD signaling pathway (30) (**Figure 1A**).

Combination therapies involving targeted agents and cytotoxic chemotherapeutics have been shown to enhance anti-tumor effects by inducing pyroptosis, thereby stimulating a robust immune response. For instance, inhibitors targeting polo-like kinase 1 (PLK1) can enhance the anti-tumor activity of cisplatin by inducing pyroptosis in esophageal squamous cell carcinoma (31). In lung cancer, small molecule inhibitors targeting KRAS, EGFR, or ALK can trigger apoptosis via the mitochondrial pathway and induce pyroptosis through the caspase-3-GSDME pathway (32). Additionally, research has demonstrated that targeting kinases such as BRAF and MEK, as well as activating the transcription factor p53, can induce pyroptosis in melanoma and non-small cell lung cancer, respectively (33, 34). These findings highlight the potential of inducing pyroptosis as a strategy for molecularly targeted anti-tumor therapies. Moreover, compounds such as L61H10, miltirone, pyridoxine and metformin have been identified as capable of targeting key molecules in pyroptotic pathway in tumor cells, contributing to the maintenance of anti-tumor treatment efficacy while exhibiting fewer side effects (35). This promising avenue warrants further investigation.

In recent years, the role of pyroptosis in various diseases has garnered significant attention, leading to the development of therapeutic strategies targeting pyroptotic pathways. Generally, pyroptosis plays opposing roles in inflammatory and oncological diseases. In the context of inflammatory diseases, the goal is often to inhibit pyroptotic pathways to mitigate the inflammatory response. Conversely, in the treatment of malignant tumors, the activation of pyroptosis is desired to induce cell death in tumor cells. Therefore, targeting and inducing pyroptosis presents a novel therapeutic approach, particularly for tumors with high expression of molecules such as GSDMD and GSDME. However, it is essential to note that therapeutic strategies aimed at targeting pyroptosis require further investigation and evaluation through various clinical trials.

## Necroptosis

Necroptosis is a form of regulated necrosis that shares morphological characteristics with necrosis (36). It was first observed in 1996 in porcine kidney cells infected with vaccinia virus, which expresses CrmA protein that inhibits both CASP1 and CASP8 (37). CASP8 was found to play a crucial role in negatively regulating this form of cell death (38). Necroptosis typically occurs when CASP8 is inhibited, either genetically or through caspase inhibitors such as Z-VAD-FMK (39). The activation of receptors like TNFR, FAS, TLR3, and ZBP1 has been associated with necroptosis induction (40).

At the molecular level, receptor-interacting serine/threonine kinase 1 (RIPK1) was initially identified as a key regulator of necroptosis (41). Subsequently, receptor-interacting serine/threonine kinase 3 (RIPK3), a downstream effector of RIPK1, was found to critically modulate necroptosis mediated by death receptors (42, 43). RIPK3 controls the phosphorylation of downstream molecule MLKL, which has been shown to function as the executioner of necroptosis. The phosphorylation cascade involving RIPK1, RIPK3, and MLKL, as well as the formation of the necrosome, represents the canonical pathway for necroptosis induction (44, 45).

The role of necroptosis in cancer remains ambiguous, with evidence suggesting it can either suppress or promote tumor progression. In most of the cases, necroptosis occurs when apoptotic signaling is impaired, allowing it to act as a barrier to tumor growth (46). However, necroptosis also triggers inflammatory responses that could contribute to tumor promotion (47). For instance, key necroptotic proteins, such as RIPK3, are often downregulated in various cancers, and patients with low RIPK3 expression generally have poorer prognosis compared to those with higher expression (48, 49). Downregulation of RIPK1 has been observed in head and neck squamous cell carcinoma, with its expression correlating with disease progression (50). In contrast, many cancers demonstrate upregulation of necroptotic factors. For instance, in pancreatic ductal adenocarcinoma, elevated levels of RIPK1, RIPK3, and MLKL are associated with accelerated tumor progression (51). Similarly, in breast cancer models, the absence of RIPK1, RIPK3, and MLKL results in slower tumor growth and heightened sensitivity to radiotherapy (52). Interestingly, necroptosis has also been implicated in anti-tumor immunity, with research suggesting that RIPK3 plays a regulatory role in the activity of natural killer T (NKT) cells, enhancing NKT-mediated anti-tumor responses (53). Furthermore, studies elucidated that targeting necroptosis can enhance antitumor immunity by activating antigen-presenting cells, promoting cross-priming of CD8+ T cells, and triggering antitumor immune responses (54).

Although necroptosis can play a dual regulatory role in tumor development, inducing or modulating necroptosis presents a promising strategy for bypassing apoptosis resistance in treatment-resistant tumors under certain conditions. An increasing number of compounds have been found to induce necroptosis. For instance, shikonin, a natural compound, has been shown to bypass drug resistance by inducing necroptosis via the RIPK1/RIPK3-dependent pathway (55). Similarly, the classic chemotherapeutic agent 5-fluorouracil (5-FU) can suppress tumor cells through a TNF-dependent necroptotic pathway when caspase activity is inhibited (56). Recently, researchers have also reported that Z-DNA-mediated necroptosis can be induced in liver cancer cells by the anti-cancer compound CBL0137 (57). As well as the methylated indolequinone, MAC681 has demonstrated antileukemic potential through the induction of immunogenic necroptosis and PARP1 degradation (58). In addition, small-molecule compounds such as cryptotanshinone (CPT) have also been identified as necroptosis inducers in lung cancer (59) (**Figure 1B**). By the way, death receptor ligands, some viruses, and even radiotherapy have been shown to suppress tumor growth, at least in part, by inducing necroptosis (60).

Taking together, increasing evidence suggests that necroptosis exhibits complex interactions with tumor immunity, autophagy, and apoptosis, playing a significant role in tumor progression, metastasis, immune surveillance, and patient prognosis. Targeting necroptosis has emerged as a potential novel strategy in cancer treatment, enhancing the sensitivity of anti-tumor therapies and supporting immunotherapeutic approaches.

## Cuproptosis

Cuproptosis is a novel form of regulated cell death triggered by intracellular copper accumulation. It is driven by the binding of excess copper to mitochondrial lipoylated proteins, disrupting their structure and leading to the aggregation of toxic protein complexes. This disruption destabilizes mitochondrial function, causing proteotoxic stress that leads to cell death. The process is regulated by key molecules like Ferredoxin 1 (FDX1) and lipoic acid synthase (LIAS), which cause aggregation of lipoylated TCA enzymes such as dihydrolipoamide acetyltransferase (DLAT). Meanwhile, FDX1 also induces the transform of Cu^2+^ to Cu^+^, which leads to the binding and destabilization of mitochondrial iron-sulfur (Fe-S) cluster proteins (61). Increasing evidence suggests that cuproptosis is associated with mitochondrial dysfunction. Excessive mitochondrial copper ion concentrations can lead to structural and functional damage to mitochondria (62). Saris et al. reported that copper overload in rats disrupts mitochondrial membrane potential, induces mitochondrial swelling, oxidative stress, and calcium efflux (63). Yang et al. found that excessive copper ions attack mitochondrial protein thiols, impairing mitochondrial defense systems, leading to a decrease in mitochondrial membrane potential and ATP levels. Copper ion-derived free radicals can directly oxidize sulfhydryl residues in respiratory chain complex IV on the inner mitochondrial membrane, thereby inhibiting its activity (64). Zischka et al. demonstrated that excessive copper ions directly attack cysteine residues in the mitochondrial inner membrane, altering the conformation and activity of inner membrane proteins and affecting mitochondrial oxidative phosphorylation (65). Brancaccio et al. showed that excessive copper disrupts the assembly and maturation of iron-sulfur cluster proteins in the mitochondrial respiratory chain (66). Steverding et al. suggested that lipid peroxidation products, such as alkenes or aldehydes caused by copper overload, might interact with numerous lysine residues on respiratory chain complexes, altering their conformation and charge (67). Liao et al. observed that copper overload affects mitochondrial metabolism, leading to decreased mitochondrial membrane potential, increased membrane permeability, and induction of mitochondria-related apoptosis in renal cells (68). These findings collectively indicate that mitochondrial copper overload damages mitochondrial structure and function.

In cancer biology, cuproptosis is particularly relevant as some tumors exhibit increased susceptibility to copper-induced toxicity. This presents opportunities for developing targeted therapies by modulating copper levels to selectively induce cell death in tumor cells (69).

Current investigations into therapeutic strategies suggest that combining disulfiram and copper (DSF/Cu) with standard chemotherapy could be an effective cancer treatment approach (70). Furthermore, research indicates that triptolide can also induce cuproptosis, presenting a novel antitumor strategy for cervical cancer by specifically targeting the X-Linked inhibitor of apoptosis (XIAP) (71) (**Figure 1C**). However, further research is needed to fully elucidate the pathways involved and optimize the therapeutic strategies targeting this form of cell death.

Nanoparticles (NPs) have emerged as promising tools for inducing cuproptosis. Research has reported the development and comparison of two diethyldithiocarbamate-copper oxide nanocomplexes (DC), DC(I + II) NPs (diethyldithiocarbamate (DD) nanocomplex combined with Cu_4_O_3_) and DC(I) NPs (DD nanocomplex combined with Cu_2_O), in combination with DD, for the treatment of metastatic liver cancer. DC (I + II) NPs showed superior efficacy by selectively inducing cuproptosis, disrupting mitochondrial enzymes, and suppressing cancer stemness and metastasis markers, while maintaining normal liver function and hematological parameters. These findings establish DC (I + II) NPs as a highly effective therapeutic formulation for metastatic liver cancer (72). Study has demonstrated novel nanocomplexes of diethyldithiocarbamate (DE) with copper oxide (CD NPs) and zinc oxide (ZD NPs) NPs to target cancer stem cells and disrupt redox balance in metastatic breast cancer. CD NPs demonstrated superior efficacy by selectively inducing oxidative stress, inhibiting ALDH1A, reducing tumor size, and eradicating liver metastases, making them a promising and safe nanomedicine for metastatic breast cancer treatment (73). Abu-Serie et al. developed novel nanoformulations of copper diethyldithiocarbamate by chelating diethyldithiocarbamate to bacterially and chemically synthesized copper oxide NPs. The chemically synthesized nanoformulation demonstrated superior anticancer efficacy compared to biosynthesized CD NPs, with higher cellular uptake, stronger ALDH1A1 inhibition, and enhanced free radical generation, making it a promising candidate for further investigation in animal models (74).

## Ferroptosis

Ferroptosis was first identified and described during a compound screening, where the compound erastin was found to induce this novel form of non-apoptotic regulated cell death in certain cell lines (75, 76). Ferroptosis differs from other forms of regulated cell death in several ways. Morphologically, cells undergoing erastin-induced ferroptosis exhibit abnormalities such as mitochondrial shrinkage, reduced cristae, and outer membrane condensation and rupture (77). This process may be regulated by pro-apoptotic BCL2 family members such as BID and PUMA (78). Mechanistically, ferroptosis is distinct from apoptosis and necroptosis, characterized by iron-catalyzed lipid peroxidation driven by Fenton reactions and lipoxygenases. Polyunsaturated fatty acids (PUFAs) in membrane lipids are the primary targets of lipid peroxidation (79, 80).

The exact mechanism by which uncontrolled lipid peroxidation triggers ferroptosis remains incompletely understood. Molecular dynamics studies have suggested that lipid peroxidation induces membrane thinning, which facilitates the penetration of oxidative agents into the cell, creating a self-perpetuating cycle that destabilizes the plasma membrane and ultimately leads to pore formation and rupture (81). Glutathione peroxidase 4 (GPX4) is a crucial regulator in this process, protecting cellular membranes from oxidative damage and acting as a key inhibitor of ferroptosis. Ferroptosis is frequently linked to the downregulation or inhibition of GPX4. Thus, ferroptosis represents a distinct form of regulated cell death, intricately associated with oxidative stress and lipid peroxidation.

Ferroptosis inducers are broadly classified into two main categories (1): Direct inducers of lipid peroxidation: such as RSL3 (82) and ML162 (83), inhibit glutathione peroxidase 4 (GPX4), leading to the accumulation of ROS within cells. This process is iron-dependent. (2) Indirect inducers that deplete cellular antioxidant defenses, such as erastin, which can directly bind to the Xc- system (SLC7A11-SLC3A2 complex), blocking the transport of cystine into cells, leading to the accumulation of lipid peroxides and ultimately inducing ferroptosis (84). Additionally, inducers like FIN56 (C_25_H_31_N_3_O_5_S_2_) promote GPX4 degradation (85), while FINO2 (C_15_H_28_O_3_) generates ROS to accelerate lipid peroxidation (86).

Ferroptosis was initially identified and characterized in RAS-mutant cancer cells, many of which exhibit sensitivity to this form of cell death. However, tumor cells from different tissue origins show varying levels of sensitivity to ferroptosis (78). For instance, studies have demonstrated that artesunate can induce ferroptosis in glioblastoma cells via p38-ERK pathway (87), and diffuse large B-cell lymphoma (DLBCL) cells are highly sensitive to erastin-induced ferroptosis (88). In glioblastoma treatment with temozolomide, ferroptosis triggered by DMT1-dependent pathway has been identified as a key mechanism of tumor cell death (89). Additionally, researchers have also identified that targeting AKT kinase with MK2206 induces ferroptosis in colorectal cancer by modulating FTO/YTHDF2-dependent m6A methylation of GPX4, resulting in its upregulation and subsequent degradation (90) (**Figure 1D**). As a distinct form of regulated cell death, ferroptosis holds potential for treating tumors resistant to apoptosis-inducing agents.

Interestingly, cancer cells that have undergone epithelial-to-mesenchymal transition (EMT) tend to accumulate more polyunsaturated fatty acids (PUFAs), the substrates of lipid peroxidation and ferroptosis. ZEB1, a key player in both adipogenesis and EMT, acts as a mechanistic bridge in this process (91). This makes mesenchymal-like cancer cells more reliant on the protective function of GPX4 (92). *In vitro* studies have shown that targeting GPX4 can induce ferroptosis in chemotherapy-resistant cells, highlighting the therapeutic potential of the ferroptosis pathway in treating drug-resistant cancers (93). Besides, reports indicate that targeting hypoxia-inducible factor 1 alpha (HIF1A), yes-associated protein (YAP), the activating transcription factor (ATF) protein family, and p53 can lead to the accumulation of ROS, ultimately triggering ferroptosis (94). Additionally, tyrosine kinase inhibitors (TKIs) have been extensively utilized in targeted and precision medicine, however, the development of drug resistance remains a significant challenge in their therapeutic efficacy. Studies suggest that targeting ferroptosis-related pathways may enhance anticancer activity and offer promising strategies for overcoming TKI resistance (95).

NPs have emerged as promising tools for inducing ferroptosis. Abu-Serie et al. developed and evaluated nanoformulations of diethyldithiocarbamate (DDC) with ferrous oxide NPs (DFeO NPs) and ferric oxide NPs (DFe_2_O_3_ NPs), demonstrating their ability to induce ferroptosis and oxidative stress, effectively eradicate cancer stem cells, and reduce metastatic activity without causing adverse effects *in vivo (* 96). Additionally, Abu-Serie et al. demonstrated that the unique nanocomplexes (DE-FeO NPs) of diethyldithiocarbamate (DE, an ALDH1A1 inhibitor) with ferrous oxide NPs (FeO NPs) exhibit superior performance compared to standard chemotherapy in attenuating chemoresistance and radioresistance in glioblastoma by increasing lipid peroxidation and ROS while depleting glutathione and glutathione peroxidase 4 (97). Abu-Serie developed a nanocomplex of FeO NPs and diethyldithiocarbamate (FD) and demonstrated that its combination with 5-fluorouracil effectively induces ferroptosis, reduces cancer stem cell populations, and suppresses metastasis, showcasing strong synergistic anticancer effects (98). Abu-Serie developed a nanocomplex of ferrous oxide NPs (F(II) NPs) and diethyldithiocarbamate (DE) (DF(II) NPs) to induce selective ferroptosis for treating metastatic liver cancer. DF(II) NPs demonstrated superior therapeutic efficacy and safety compared to the typical DF(II) complex, effectively eradicating metastatic liver cancer cells by enhancing lipid peroxidation, suppressing antioxidant defenses, and downregulating oncogenic and cancer stem cell genes in both *in vitro* and *in vivo* models (99).

Nevertheless, further investigation is essential to identify the malignancies most sensitive to ferroptosis and to determine the appropriate ferroptosis inducers for specific cancer therapies. Additionally, understanding the relationships and distinctions between ferroptosis and other forms of regulated cell death in various pathological contexts is critical. This knowledge could significantly contribute to optimizing therapeutic strategies and enhancing the efficacy of cancer treatments.

## Disulfidptosis

Disulfidptosis is a newly identified form of RCD triggered by abnormal disulfide bond formation, leading to cytoskeletal collapse, particularly in actin filaments, and cell death. Mechanistically, SLC7A11 imports cysteine, and GLUT1 dysfunction impairs glucose uptake, causing disulfide stress and triggering disulfidptosis (100). It predominantly occurs in cancer cells with elevated glucose metabolism, where cysteine oxidation disrupts the cytoskeletal integrity (101). This mechanism holds particular relevance in cancer biology as it represents a novel target for therapeutic interventions, especially in glucose-dependent tumors.

**Figure 2:**
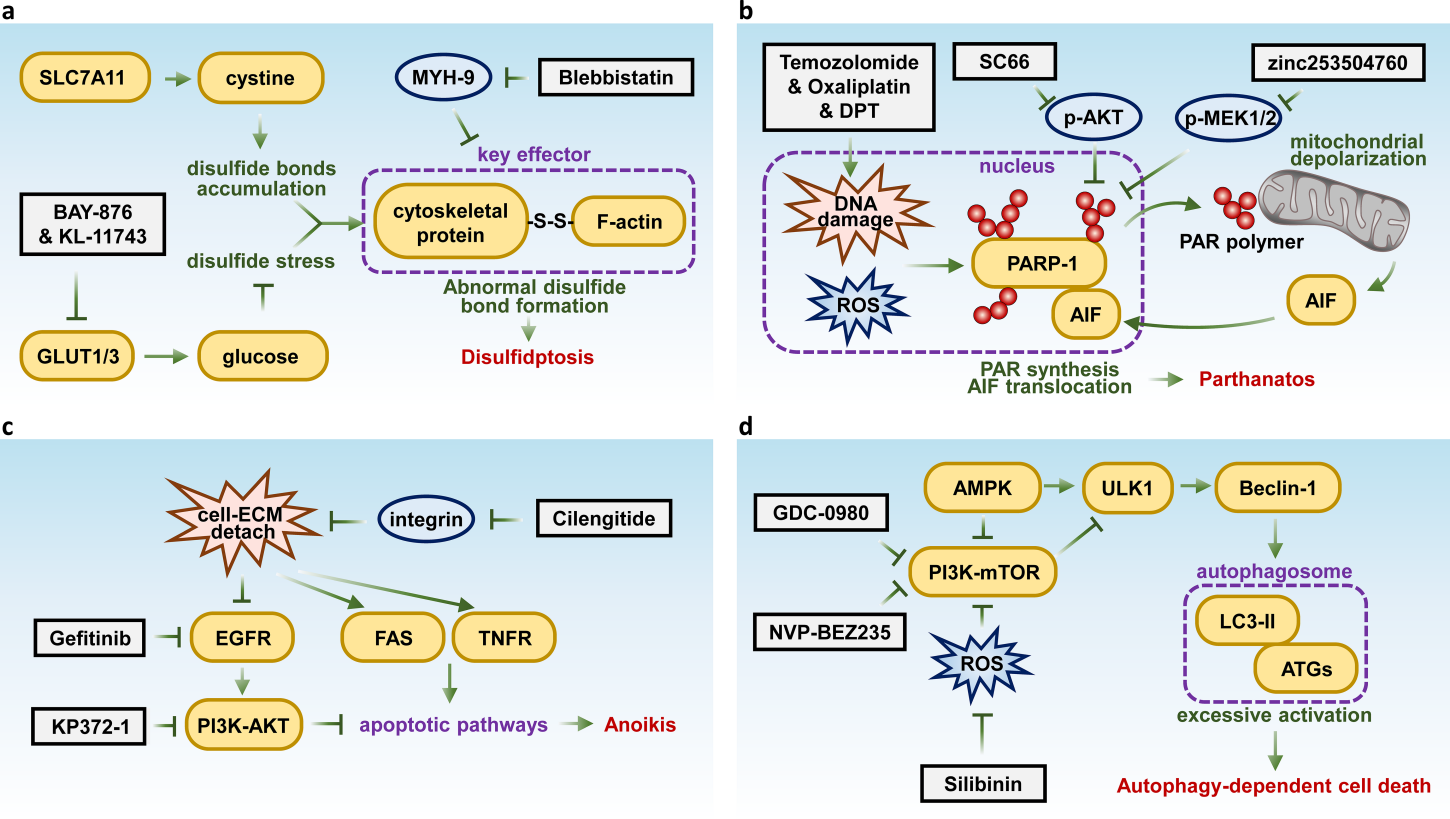
Key molecules and therapeutic targets in disulfidptosis, parthanatos, anoikis, and autophagy-dependent cell death. Schematic summarizing the key molecular pathways and therapeutic targets of **(A)** disulfidptosis, **(B)** parthanatos, **(C)** anoikis, and **(D)** autophagy-dependent cell death.

Recent studies suggest that inhibiting glucose transporters (GLUTs) may be an effective strategy for inducing disulfidptosis in SLC7A11 high expression tumors, which are common in many human cancers. For instance, the GLUT1 inhibitor BAY-876 and the GLUT1/3 inhibitor KL-11743 have been shown to induce disulfidptosis in cancer cells (102). Additionally, the MYH9 inhibitor Blebbistatin induces F-actin contraction and cell shrinkage, mimicking disulfidptosis-like changes, thus enhancing drug sensitivity in liver cancer (103) (**Figure 2A**). These findings underscore the potential of disulfidptosis-targeted therapies in treating aggressive and resistant malignancies.

## Parthanatos

Parthanatos, also known as poly(ADP-ribose) polymerase 1 dependent cell death (PARP1-dependent cell death), is a form of regulated cell death that can be activated under conditions such as oxidative stress that induce high levels of DNA damage (104, 105). Unlike apoptosis, PARP1-dependent cell death does not involve apoptotic bodies or DNA fragmentation, nor does it exhibit cellular swelling. Instead, it is characterized by distinct plasma membrane rupture (106, 107). Mechanistically, the process requires hyperactivation of PARP1. PARP1 recognizes DNA damage and initiates the formation of poly(ADP-ribose) (PAR) polymers using nicotinamide adenine dinucleotide (NAD) and ATP. On one hand, this synthesis depletes cellular ATP and NAD, while on the other hand, it causes mitochondrial inner membrane depolarization and the release of apoptosis-inducing factor (AIF) (105, 108). AIF then translocates to the nucleus, where it induces chromatin condensation and large-scale DNA fragmentation, leading to chromatin dissolution, a hallmark of parthanatos (109). Meanwhile, AIF-independent parthanatos has been reported, where PARP-1 activation leads to cell death via mitochondrial dysfunction and energy collapse in response to H_2_O_2_, without AIF involvement in the execution of cell death (110).

Parthanatos has been implicated in the pathogenesis of various diseases, including retinal detachment, Parkinson’s disease, smoking-related lung disease, ischemic stroke, and oxidative stress-induced hearing loss (111–115). In the context of cancer, multiple molecules within the parthanatos pathway are intricately linked to tumorigenesis and progression. PARP1 plays a crucial role in DNA damage repair, it can facilitate DNA repair and replication in certain contexts, promoting cell survival, while in other situations, it may induce DNA breaks that lead to cell death. Studies have indicated that tumors tend to develop more rapidly in the absence of PARP1 (116). Additionally, PARP1 has been demonstrated to inhibit tumor proliferation and metastasis (117). Patients exhibiting negative to low expression levels of PARP1 tend to have poorer prognoses and shorter overall survival (118). Interestingly, a crucial aspect of parthanatos is the catalytic activation of PARP1, though during apoptosis, activated caspase-3 cleaves and inactivates PARP1 (119). This implies that inducing parthanatos in malignancies, particularly those with inhibited apoptotic pathways, can effectively suppress tumor growth.

Recently, several drugs and compounds have been identified that can induce parthanatos in cancer cells. Chemotherapy agents such as temozolomide and oxaliplatin have been shown to trigger parthanatos by inducing extensive DNA damage (120, 121). Furthermore, the AKT kinase inhibitor SC66 has been reported to activate parthanatos in a p53-Sirt6 dependent manner (122). Deoxypodophyllotoxin (DPT) has also been found to initiate parthanatos by promoting the nuclear translocation of AIF via activation of mitochondrial respiratory chain complex I (123). Besides, research has revealed that the cardiac glycoside compound ZINC253504760 can induce parthanatos in multidrug-resistant (MDR) leukemia cells (124) (**Figure 2B**). Notably, reagents that promote the generation and accumulation of ROS may hold significant potential for inducing Parthanatos in cancer cells, as ROS can stimulate the formation of PAR, which initiate parthanatos (125).

Thus, from a therapeutic standpoint, further investigation into the precise mechanisms of PARP1-dependent cell death, alongside exploration of the clinical efficacy and safety of PARP1-targeted therapies, holds significant potential. Such research could offer valuable strategies for treating various malignancies, especially in cases of drug resistance, recurrence, or refractory tumors, providing a promising avenue for improving patient outcomes.

## Anoikis

Anoikis is a specific form of cell death triggered by the loss of cell contact with the extracellular matrix (ECM) or neighboring cells. Detachment of integrins deactivates survival signaling pathways, such as EGFR-PI3K-AKT, while activating apoptotic pathways, including ligand-mediated signals (e.g., TNF/TNFR, FasL/Fas) and mitochondrial pathways (126). Although anoikis shares downstream apoptotic mechanisms, it is uniquely induced by cell-ECM detachment. This specialized process is essential for maintaining tissue integrity and preventing metastasis by eliminating displaced cells. However, tumor cells that evade anoikis can survive detachment from the primary site, enabling distant metastasis (127).

Anoikis resistance is pivotal in facilitating metastasis, making it a promising therapeutic target in cancer treatment. Targeting key molecules involved in this process has shown potential to induce anoikis. For instance, the EGFR inhibitor gefitinib has been demonstrated to trigger anoikis in cervical cancer (128), while the integrin inhibitor cilengitide promotes atypical anoikis in glioma (129). Additionally, an AKT inhibitor, KP372-1 has been shown to induce anoikis in squamous cell carcinoma of the head and neck (130) (**Figure 2C**).

## Autophagy-dependent cell death

Autophagy-dependent cell death is driven by intracellular catabolic pathways regulated by over 40 autophagy-related genes and proteins (ATGs) (40). These pathways lead to excessive activation of autolysosomes, resulting in the degradation of essential cellular components and cell death. Typically, autophagy functions as a dynamic recycling system that maintains cellular homeostasis, often acting as a survival mechanism. However, recent evidence suggests that autophagy can also function as a primary mechanism of cell death, including tumor suppression (131).

Targeting key autophagy-regulating genes such as PI3K and mTOR with inhibitors like NVP-BEZ235 and GDC-0980 has shown potential to enhance the effectiveness of treatment in malignant pleural mesothelioma (132). Preclinical and clinical evidence also indicate that the autophagy inhibitor chloroquine can sensitize prostate cancer cells to treatment (133). Additionally, the natural compound silibinin has been found to induce autophagy-dependent cell death in glioma, mediated by oxidative stress and the nuclear translocation of AIF (134) (**Figure 2D**).

## Entosis

Entosis is a cellular process in which one living cell engulfs another, forming a cell-in-cell (CIC) structure. This process is initiated by cadherin/β-catenin-mediated cell adhesion and driven by actomyosin contraction regulated by Rho GTPases (135, 136). This phenomenon is often observed in cancer and plays a role in tissue homeostasis (137, 138). The engulfed cell may undergo internalization and potential degradation through LC3-associated phagocytosis (LAP) (139). As a form of cell death linked to autophagy activation, entosis involves one cell engulfing and lysing another, distinct from autophagy-dependent cell death, which entails self-destruction through autophagy. Entosis has been reported to facilitate the death of entotic cancer cells, functioning as a tumor-suppressive mechanism. However, studies also indicate that most of the tumors exhibiting the entotic phenotype tend to be more malignant and are associated with poorer prognosis, suggesting tumor cells might use mitosis as shields to evade elimination (140).

**Figure 3:**
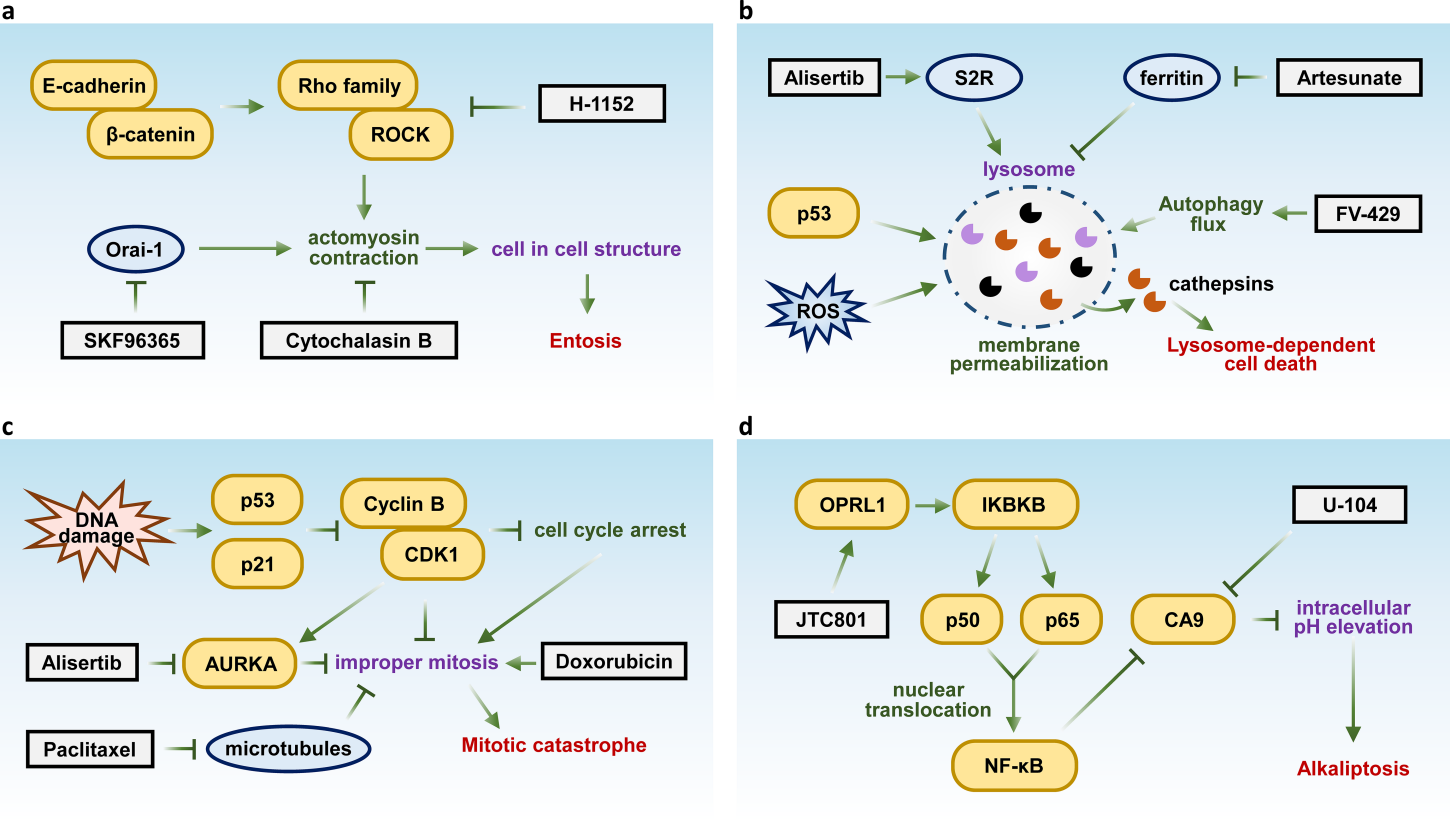
Key molecules and therapeutic targets in entosis, lysosome-dependent cell death, mitotic catastrophe, and alkaliptosis. Schematic summarizing the key molecular pathways and therapeutic targets of **(A)** Entosis, **(B)** lysosome-dependent cell death, **(C)** mitotic catastrophe, and **(D)** alkaliptosis.

Inhibition of entosis holds the potential to enhance the effectiveness of cancer therapies by sensitizing tumor cells to treatment, potentially overcoming resistance mechanisms and improving therapeutic outcomes. Recent studies have demonstrated that the inhibition of the Orai1 Ca²^+^ channel with the inhibitor SKF96365 effectively prevents entosis (141). Additionally, targeting Rho-ROCK signaling using the ROCK inhibitor H-1152 attenuates entosis by reducing actomyosin contraction (136). Furthermore, direct inhibition of actomyosin with Cytochalasin B has also been shown to suppress entosis (142) (**Figure 3A**).

## Lysosome-dependent cell death

Lysosome-dependent cell death (LDCD) is a form of programmed cell death initiated by lysosomal membrane permeabilization following to stress like p53 activation and ROS, resulting in the release of hydrolytic enzymes, such as cathepsins, into the cytosol. These enzymes facilitate cellular degradation and lead to cell death (143). Researchers also suggest that LDCD is involved in inducing apoptosis, necrosis, entosis, pyroptosis and ferroptosis (144, 145).

LDCD plays a crucial role in both neurodegenerative diseases and cancer, regulating cell death pathways and presenting promising therapeutic targets. For instance, the sigma-2 receptor (S2R) agonist siramesine triggers LDCD in breast cancer by destabilizing lysosomes and releasing cathepsins (146). Additionally, FV-429, a synthetic flavonoid compound induces LDCD in T-cell malignancies through lysosomal dysregulation (147), while artesunate enhances lysosomal function and degradation, promoting LDCD in cancer cells (148) (**Figure 3B**).

## Mitotic catastrophe

Mitotic catastrophe is a form of regulated cell death that is initiated by DNA damage and subsequent activation of the p53/p21 pathway, leading to cell cycle arrest. Dysregulation of Cyclin B/CDK1 causes improper mitotic entry, while dysfunction of Aurora-A Kinases (AURKA) impairs spindle assembly, both of which contribute to the onset of mitotic catastrophe. Following this, caspase activation is triggered, ultimately leading to cell death. These pathways function as critical safeguards against genomic instability by ensuring that cells with mitotic errors or DNA damage are eliminated (149, 150). It serves as a protective mechanism to prevent the division of damaged cells, often triggered by DNA damage.

This process also plays a significant role in enhancing the efficacy of chemotherapy in cancer treatment, as it can induce cancer cell death (151). For example, the AURKA inhibitor Alisertib disrupts chromosome segregation, leading to mitotic catastrophe in multiple myeloma (152). Similarly, paclitaxel stabilizes microtubules, impairing chromosome segregation and inducing mitotic catastrophe in gastric cancer (153). Doxorubicin has also been shown to trigger mitotic catastrophe in hepatocellular carcinoma (154) (**Figure 3C**). Although the classification of mitotic catastrophe as a form of regulated cell death remains controversial, it plays a critical oncosuppressive role by eliminating mitosis-incompetent cells, making it a promising target for cancer therapy and an important area of study.

## Alkaliptosis

Alkaliptosis is a recently identified form of regulated cell death characterized by an increase in intracellular pH, primarily driven by the inhibition of carbonic anhydrase IX (CA9), a key enzyme regulating pH homeostasis. Inhibition of CA9 disrupts this balance, leading to the accumulation of alkaline metabolites and triggering a cascade of cellular stress responses that culminate in cell death (155). Targeting alkaliptosis has emerged as a potential therapeutic approach in cancer treatment (156). Recent studies have shown that the opioid receptor-like 1 (OPRL1) antagonist JTC801 can induce alkaliptosis by activating the NF-κB pathway. The canonical NF-κB pathway is activated by ligands (e.g., lipopolysaccharide) via the IKK complex (IKKα, IKKβ, IKKγ), leading to IκBα degradation and nuclear translocation of NF-κB subunits (p50, p65) (157). CA9 is identified as a negatively regulated target of the NF-κB pathway, with its expression downregulated upon NF-κB activation (155). Another study demonstrated that direct inhibition of CA9 using the compound U-104 effectively suppressed pancreatic ductal adenocarcinoma (PDAC) cell proliferation (158) (**Figure 3D**), further highlighting its therapeutic potential.

### Regulated cell death: a double-edged sword in cancer elimination and adaptation

Pyroptosis represents a double-edged sword in cancer. GSDMD, a key effector protein of pyroptosis, is often overexpressed in gliomas, with its expression levels increasing in parallel with the WHO grading of gliomas and negatively correlating with prognosis (159). In glioma cells treated with temozolomide (TMZ), the expression of pyroptosis markers, including GSDMD, caspase-1, and IL-1β, significantly increases, accompanied by morphological changes indicative of pyroptosis. The extent of pyroptosis positively correlates with TMZ concentration, whereas inhibiting GSDMD expression markedly reduces TMZ-induced pyroptosis and facilitates tumor cell proliferation (159, 160). These findings suggest that GSDMD plays a crucial role in modulating glioma cell sensitivity to TMZ. Similarly, 5-fluorouracil has been shown to induce caspase-3/GSDME-dependent pyroptosis in gastric cancer cells, shedding light on the mechanisms underlying chemotherapy in gastric cancer (161). Furthermore, cannabidiol triggers the integrated stress response and mitochondrial stress in hepatocellular carcinoma cells, leading to the activation of ATF4 and its downstream target CHOP. This subsequently promotes the expression of Bax, a member of the BCL-2 family, and induces caspase-3/caspase-9/GSDME-dependent pyroptosis (162).

The ability of cell death to trigger adaptive immune responses is referred to as immunogenic cell death (163). Pyroptosis, with its molecular mechanisms that induce a strong inflammatory response, is considered a form of ICD under certain conditions (18, 164). During immunogenic pyroptosis, the release of numerous tumor antigens, damage-associated molecular patterns (DAMPs), and inflammatory cytokines can efficiently drive dendritic cells (DCs) maturation, trigger activation of tumor antigen-specific T cells, facilitate cytotoxic T lymphocyte infiltration into tumors, transform immunologically “cold” tumors into “hot” tumors, improve the responsiveness to immune checkpoint blockade therapy, and ultimately strengthen the body’s antitumor immune response (165–167).

The specific role of necroptosis in tumors remains difficult to define. Hänggi et al. discovered that triggering necroptosis in established breast tumors creates a myeloid-dominated immunosuppressive microenvironment. This environment impairs T cell activity, facilitates tumor progression, and shortens survival (168). However, RIPK3, a critical molecule in the initiation of necroptosis, has been shown to suppress migration and invasion of colorectal cancer cells when overexpressed (169). Furthermore, ectopic expression of RIPK3 in cancer cells lacking its expression can inhibit tumor growth (49, 170). These findings suggest that the loss or downregulation of RIPK3 in tumor cells promotes cell survival and tumorigenesis.

Necroptosis in cancer cells holds promise for creating an inflammatory immune microenvironment within the tumor by releasing DAMPs, cytokines, and/or chemokines, which can lead to either tumor-promoting or antitumor effects (171–173). Necroptotic tumor cells attract macrophages and DCs, which are activated by DAMPs and cytokines. Activated DCs migrate to lymph nodes, where they prime naïve CD4+ and CD8+ T cells. The naïve T cells then differentiate into effector T cells, exit the lymph nodes, re-enter circulation, and infiltrate tumor tissues to exert antitumor effects. RIPK3 has been shown to induce cytokine secretion, activate NKT cells, and enhance their tumor-killing activity. However, necroptotic tumor cells can also attract myeloid-derived suppressor cells (MDSCs) and tumor-associated macrophages (TAMs), leading to tumor-associated immunosuppression.

Ferroptosis acts as a double-edged sword in regulating tumor immunity. On one hand, ferroptosis influences the phenotype and function of immune cells, while immune cells can also regulate the ferroptosis process in tumor cells. For example, activated CD8+ T cells secrete IFN-γ, which inhibits the Xc− system, ultimately inducing ferroptosis in tumor cells and exerting antitumor effects. Ferroptosis cells can release specific signals, such as arachidonic acid derivatives and the damage-associated molecular pattern protein, high-mobility group box 1 (HMGB1), to mediate antitumor immunity (174). On the other hand, ferroptosis may lead to a state of chronic inflammation closely associated with tumor initiation and progression. To support the survival of neighboring tumor cells or evade immune detection, ferroptosis tumor cells and tumor-infiltrating immune cells can produce immunosuppressive mediators, such as prostaglandin E2 (PGE2), thereby inhibiting antitumor immunity and ultimately promoting tumor growth. For instance, although inhibition of GPX4 increases intracellular lipid peroxidation products and triggers ferroptosis in tumor cells, it simultaneously enhances PGE2-mediated immune evasion, fostering tumor progression (175).

Studies have shown that CD8+ T cells and neutrophils promote ferroptosis in tumor cells through the secretion of interferon-γ (IFN-γ) and the transfer of myeloperoxidase-containing granules, respectively (176). Other components of the tumor microenvironment (TME), such as transforming growth factor-β1 (TGF-β1) and n-3 and n-6 polyunsaturated fatty acids (PUFAs), also enhance ferroptosis in tumor cells (177, 178). Subsequently, ferroptotic cancer cells release immunostimulatory signals that promote the maturation of dendritic cells, activate M1-polarized macrophages, and enhance T-cell infiltration and activity within tumors. Additionally, ferroptotic cancer cells reduce the release of TGF-β1, thereby inhibiting immunosuppressive cancer-associated fibroblasts (CAFs) (179). Furthermore, ferroptosis induction disrupts the immunosuppressive functions of various immune-suppressing cells, including tumor-infiltrating neutrophils (180), myeloid-derived suppressor cells (MDSCs) (181), regulatory T (Treg) cells (182), and M2-polarized tumor-associated macrophages (TAMs) (183), thereby enhancing antitumor immunity.

Autophagy-dependent cell death also plays a dual role in tumors. Elevated autophagy levels help tumor cells survive metabolic stress caused by starvation, hypoxia, and factor deprivation (184, 185). Additionally, enhanced autophagy enables tumor cells to resist damage from radiotherapy and chemotherapy, conferring a high level of stress tolerance. This allows tumor cells to limit damage, maintain viability, sustain dormancy, and promote recovery (186). Conversely, autophagy also plays a critical role in mitigating damage during stress responses, which may hinder tumorigenesis. By clearing damaged proteins and organelles, autophagy may help maintain energy balance through intracellular recycling and ultimately prevent genomic damage, a key driver of tumor development. Overall, autophagy equips tumor cells with the capacity to adapt and evolve under selective pressures, progressively becoming more harmful to the host. This adaptability contributes to the difficulty of effectively treating cancer (186).

Regarding the role of entosis in tumors, from the perspective of internalized cells, entosis represents a form of “self-cannibalism” among tumor cells, capable of inhibiting tumor growth by driving the death of internalized cells. However, from the perspective of host cells, entosis can promote tumor progression. On one hand, internalized cells can provide nutrients to host cells; on the other hand, entosis can disrupt host cell division, potentially leading to genomic instability and facilitating tumor progression (187). In PDAC, entosis is the predominant form of CIC and is associated with tumor invasiveness and poor prognosis. Tumor cells can exploit entosis to generate highly invasive subpopulations. Within these internalized cells, the expression of several oncogenes is upregulated, conferring enhanced tumorigenic potential in both *in vitro* and *in vivo* models (188).

Mitotic catastrophe serves as a safeguard mechanism to prevent genomic instability, limiting the proliferation of unstable cells and thereby contributing to cancer prevention (189). However, even after undergoing mitotic catastrophe, certain tumor cells can survive by evading cell death and adapting to genomic instability (189). Study has shown that p53 can mediate mitotic catastrophe. p53 deficient cells exhibit a higher frequency of polyploidization in response to mitotic inhibitors compared to their p53 proficient counterparts. Moreover, the absence of p53 permits multipolar divisions in tetraploid cells, leading to the generation of aneuploid, genomically unstable progeny, which can contribute to tumorigenesis (190, 191).

Regarding cuproptosis, it can play dual roles in tumors. On one hand, it promotes tumor proliferation, metastasis, and angiogenesis. Excess Cu^+^ can activate the MAPK-ERK pathway, thereby enhancing tumor cell proliferation (192) and stimulating the synthesis of various angiogenic factors, including angiopoietin, VEGF and FGF1 (193). Copper can also facilitate tumor metastasis through the LOX pathway (194, 195). Additionally, it may help tumor cells evade immune clearance by upregulating the expression of PD-L1 (196). On the other hand, copper overload can exert anti-tumor effects by interfering with the mitochondrial TCA cycle, depleting GSH, and reducing the antioxidant capacity of tumor cells, ultimately inducing tumor cell death (197).

Notably, cuproptosis disrupts the cell membrane, leading to the release of DAMPs that trigger a robust immune response. This process enhances lymphocyte infiltration and drives the secretion of inflammatory cytokines, effectively reshaping the immunosuppressive TME. Furthermore, the combination of ES@CuO nanoparticles with PD-1 therapy significantly boosts the antitumor effectiveness of immune checkpoint inhibitors (198).

### Cross-talk among regulated cell death pathways

Parthanatos shares some characteristics with necroptosis, apoptosis, and autophagy, but differs significantly in its molecular mechanisms. Unlike apoptosis, Parthanatos does not result in the formation of small DNA fragments or apoptotic bodies (199). In contrast to necrosis, it does not cause swelling of cellular organelles (107, 200). Unlike autophagy, Parthanatos does not involve the formation of autophagosomes or lysosomal degradation (201). Compared to necroptosis, Parthanatos does not induce swelling of the plasma membrane and organelles, cell lysis, or activation of RIPK1 (201).

Ferroptosis and pyroptosis exhibit distinct characteristics, yet both mechanisms hold significant research value in the field of cancer therapy. Studies have shown that antitumor immune cells, such as CD8+ T cells, play a dual role in promoting and inducing these two forms of cell death (202). On one hand, CD8+ T cells secrete granzyme A (GzmA), which acts as a cleavage enzyme for GSDMB. The cleaved GSDMB subsequently triggers pyroptosis. On the other hand, CD8+ T cells release IFN-γ, which downregulates SLC7A11, leading to the accumulation of lipid ROS and the induction of ferroptosis. Moreover, tumor cells undergoing pyroptosis further enhance the activation and differentiation of antitumor immune cells, contributing to the eradication of the tumor.

Some evidence suggests a crosstalk between necroptosis and pyroptosis. Necroptosis, induced through RIPK3 activation, promotes NLRP3-caspase-1-mediated IL-1β secretion (203). Subsequent experiments using MLKL and inflammasome gene knockout models further support that necroptotic signaling can trigger the RIPK3-mixed lineage kinase domain-like protein (MLKL)-NLRP3-Caspase-1 axis (204).

Inhibition of ULK1 impedes mitophagy, resulting in the accumulation of ROS. The generated ROS subsequently activates the NLRP3-Caspase3/8 signaling axis, leading to the cleavage of GSDME and the formation of GSDME-N. GSDME-N integrates into the plasma membrane, promoting pyroptosis (205).

In certain contexts, selective autophagy acts as a pro-survival mechanism during ferroptosis by selectively removing damaged or dysfunctional cellular components, thereby limiting lipid peroxidation and maintaining cellular homeostasis. For instance, endoplasmic reticulum (ER)-phagy specifically targets and degrades portions of the ER. The ER-resident receptor RETREG1/FAM134B interacts with MAP1LC3 to facilitate ER degradation through autophagy. In the context of ferroptosis, ferroptosis inducers effectively activate RETREG1-mediated ER-phagy, thereby suppressing ferroptosis. However, when RETREG1 is knocked down, ER-phagy is inhibited, leading to increased sensitivity to ferroptosis (206). Simultaneously, ACSL4 facilitates the formation of lipid peroxidation substrates during ferroptosis. The ACSL4 protein contains six KFERQ-like motifs, making it a substrate for chaperone-mediated autophagy (CMA). CMA-mediated degradation of GPX4 promotes ferroptosis, whereas CMA-mediated degradation of ACSL4 can suppress this process (207).

Autophagy-dependent cell death, entosis, and lysosome-dependent cell death are closely linked to autophagic flux, regulated by key molecules such as AMPK activation and mTOR suppression. These processes lead to lysosomal membrane permeabilization and hydrolase release, resulting in cell death. Additionally, they share common upstream signals, including p53 activation and ROS accumulation (208, 209).

Cuproptosis and ferroptosis share critical cross-talk in regulating cell death pathways. Both involve mitochondrial dysfunction, with cuproptosis driven by copper-induced TCA cycle protein aggregation and ferroptosis triggered by lipid peroxidation from ROS accumulation. Mitochondrial metabolism links the two pathways, as disruption of iron-sulfur cluster biogenesis and reactive oxygen species production influences both. Furthermore, gene interactions between cuproptosis regulators (e.g., FDX1, DLAT) and ferroptosis regulators (e.g., GPX4, SLC7A11) emphasize their interaction, highlighting potential therapeutic strategies that target mitochondrial vulnerabilities in cancers (210).

## Summary

RCD is fundamental to disease pathology, with numerous studies linking its dysregulation to a wide range of conditions. Overactivation of specific cell death pathways can result in pathological cell death, contributing to neurodegenerative diseases such as Alzheimer’s. Conversely, suppression of these pathways can facilitate abnormal cell proliferation, leading to tumorigenesis. Identifying aberrant RCD pathways in various diseases, particularly cancers, and developing targeted therapies for these pathways presents promising potential for novel treatments. This review also highlights drugs that induce distinct RCD forms and their molecular targets (**Figures 1**–**3**).

Inducing RCDs in cancer therapy presents significant challenges, primarily due to the need for targeting specific pathways while minimizing harm to healthy tissue. Cancer cells often exhibit resistance to RCDs through altered signaling or evasion of death pathways, particularly apoptosis. Future therapeutic strategies include developing targeted therapies that selectively activate RCD pathways like pyroptosis, ferroptosis, or necroptosis, as well as leveraging nanomedicines. Combining RCD inducers with immunotherapies could enhance therapeutic efficacy. Non-apoptotic RCDs, which bypass apoptotic resistance, offer promising approaches for overcoming drug resistance in cancer treatment. Additionally, certain RCD types induce immunogenic cell death, stimulating anti-tumor immune responses. This provides a new avenue for integrating RCD induction with immunotherapy to improve treatment outcomes. However, the mechanisms of some RCD types remain poorly understood, the activation of some RCDs might be double-edged swords for eliminating cancer cells, highlighting the need for further research. Exploring novel RCD pathways through clinical trials will be critical for developing innovative and effective cancer treatments that improve patient outcomes.
